# Differential MicroRNA Expression in Human Macrophages with *Mycobacterium tuberculosis* Infection of Beijing/W and Non-Beijing/W Strain Types

**DOI:** 10.1371/journal.pone.0126018

**Published:** 2015-06-08

**Authors:** Lin Zheng, Eric Leung, Nelson Lee, Grace Lui, Ka-Fai To, Raphael C. Y. Chan, Margaret Ip

**Affiliations:** 1 Department of Microbiology, The Chinese University of Hong Kong, Hong Kong SAR, China; 2 Department of Medicine & Therapeutics, The Chinese University of Hong Kong, Hong Kong SAR, China; 3 Department of Anatomical & Cellular Pathology, The Chinese University of Hong Kong, Hong Kong SAR, China; Public Health Research Institute at RBHS, UNITED STATES

## Abstract

**Objectives:**

The role of microRNAs in association with *Mycobacterium tuberculosis *(MTB) infection and the immunology regulated by microRNAs upon MTB infection have not been fully unravelled. We examined the microRNA profiles of THP-1 macrophages upon the MTB infection of Beijing/W and non-Beijing/W clinical strains. We also studied the microRNA profiles of the host macrophages by microarray in a small cohort with active MTB disease, latent infection (LTBI), and from healthy controls.

**Results:**

The results revealed that 14 microRNAs differentiated infections of Beijing/W from non-Beijing/W strains (P<0.05). A unique signature of 11 microRNAs in human macrophages was identified to differentiate active MTB disease from LTBI and healthy controls. Pathway analyses of these differentially expressed miRNAs suggest that the immune-regulatory interactions involving TGF-β signalling pathway take part in the dysregulation of critical TB processes in the macrophages, resulting in active expression of both cell communication and signalling transduction systems.

**Conclusion:**

We showed for the first time that the Beijing/W TB strains repressed a number of miRNAs expressions which may reflect their virulence characteristics in altering the host response. The unique signatures of 11 microRNAs may deserve further evaluation as candidates for biomarkers in the diagnosis of MTB and Beijing/W infections.

## Introduction

Tuberculosis is one of the most common causes of death from infectious diseases. Studies have shown that one-third of the world’s population is infected with *M*. *tuberculosis* (MTB). The people who are infected with MTB but who do not have active tuberculosis have latent infection (LTBI), and they have a 10% lifetime chance that they will progress to having the active disease.

Macrophages play a key role in the immune defence, and in particular, early clearance of MTB. MTB invade and replicate within alveolar macrophages. They evade the host defence system by blocking the formation of the apoptotic envelope [1] or inhibiting plasma membrane repair [2], which lead to macrophage necrosis and dissemination of infection in the lung.

MicroRNAs (miRNAs) are small, non-coding RNAs that have an important regulatory role in gene expression programs [3]. Each miRNA has the potential to repress the expression of hundreds of genes [4]. Disease-associated miRNAs represent a new class of diagnostic marker or therapeutic targets [5]. Several of these have recently been demonstrated to regulate the components of important inflammation signalling pathways under the challenge of specific MTB antigens [6–12]. For example, miR-144* were over-expressed in the T cells of active TB patients [6], miR-146a regulating IL-6 production in dendritic cells [7]. High miR-125b expression and low miR-155 expression with correspondingly low TNF production regulate the macrophage inflammatory response [9–10], while the miR-155/miR-155* ratio was increased in PBMCs of MTB patients [12].

The effect of miRNA expression on the infection of various MTB strain types is as yet unknown. While most studies used laboratory strains, clinical strains such as that of the Beijing/W family have been associated with outbreaks and multidrug resistance, and may harbour a genetic advantage for disease. We hypothesized that miRNAs have a role in regulating the unique gene expression of macrophages in a strain- and host-dependent way. In this study, we examined the expression of 384 unique human-specific and widely expressed miRNAs from PMA-treated THP-1 derived macrophages infected with different clinical MTB strains. The results revealed unique signatures that differentiated infections of Beijing/W from non-Beijing/W strains. In addition, we also revealed that differentially expressed miRNA profiles of macrophages of patients with active MTB infection differed from those of LTBI patients and healthy controls. Pathway analyses suggested that cell membrane and extracellular matrix metabolite involve glycosaminoglycan biosynthesis and fatty acid biosynthesis; and that immune-regulatory interactions involving TGF-β signalling pathway take part in the dysregulation of critical TB processes in the macrophages. These miRNAs profiles may serve as disease-associated markers and enhance our understanding in the host-bacterial interactions in MTB infections.

## Materials and Methods

### Bacterial Strains

Twelve clinical isolates of MTB, including six Beijing/W, six non-Beijing/W strains previously isolated from patients at the Prince of Wales Hospital, Hong Kong were examined. The phenotypes and genotypes of these strains were respectively confirmed by MIC and DTM-PCR methods, as described by Chen *et al*. [13]. Briefly, DTM-PCR used three primers in a multiplex PCR to target the RD105 deletion in Beijing/W genotypes and produced a 1,466 bp product for the non-Beijing genotype and a 761 bp for the Beijing/W genotype.

### Patient recruitment and characteristics

Participants were recruited from the Prince of Wales Hospital, Hong Kong. All participants were older than 18 years and gave written informed consent. Patients who were pregnant, immune-suppressed, or who had diabetes or autoimmune disease were excluded. From each individual in the three cohorts: the healthy (n = 3), the latent (n = 4), and the active TB patients (n = 3), whole blood specimens were collected for monocytes isolation. Patients with active TB were confirmed by a positive acid-fast smear and culture. Active TB patients were prospectively recruited and sampled before any anti-mycobacterial treatment was started. LTBI cases were identified to be positive in the IFN-γ release assay (IGRA) but without their having signs and symptoms of active disease. Healthy controls were volunteers who were excluded from any known acute or chronic infections and who were negative by IGRA. Ethics approval was obtained from the Joint Chinese University of Hong Kong, New Territories East Cluster Clinical Research Ethics Committee. All participants were older than 18 years and gave written informed consent.

### IFN-γ release assay (IGRA) testing

The QuantiFERON TB-Gold Test (Cellestis) was performed in accordance with the manufacturer’s instructions.

### PBMC isolation from whole blood

PBMCs were freshly harvested from the patients’ whole blood by using the Ficoll-Hypaque column (GE healthcare) in accordance with manufacturer’s instructions. The supernatant containing the autologous donor-specific plasma was saved and heat inactivated at 56°C for 30 min. The PBMC was resuspended in ice-cold monocyte adhesion medium (RPMI1640 + 7.5% autologous plasma, 1% penicillin-streptomycin) and incubated in a petri dish for 90minutes at 37°C. The adherent monocytes were washed with warm RPMI medium several times to remove loosely attached cells. The monocytes were detached by incubation with PBS containing 5 mM EDTA for 10–20 minutes at room temperature and were collected by centrifugation. The differentiation into macrophages was according to protocol previously described [14]. The monocytes were refed by fresh medium every 2 days and allowed to differentiate into macrophages for 10 days in vitroRNA of macrophages was harvested and kept for downstream TaqMan miRNA array experiments.

### Infection of macrophages

THP-1 cells were maintained in RPMI 1640 (Gibco,Carlsbad, CA) supplemented with 10% fetal bovine serum (Gibco). Cells were incubated with phorbolmyristate acetate (5ng/ml PMA; Sigma-Aldrich, St Louis, MO) for 48 hours to induce differentiation into a macrophage phenotype [15]. MTB isolates were cultured in Middlebrook7H9 (BD Biosciences) at 37°C, 5% CO_2_ until the cultures reached McFarland 1 (about 10^7^ CFU/mL). The MTB cells were harvested by centrifugation and the pellet was resuspended in RPMI medium and added to the macrophages. Macrophages were infected at a multiplicity of infection (MOI) of 3 bacilli/cell for 2 hours, and the excess free-floating bacilli were removed by washing the culture with fresh RPMI containing 10μg/ml gentamicin. The culture was incubated in a fresh RPMI medium without antibiotics at 37°C, 5% CO_2_for 72-hours. Uninfected control cultures of THP-1 or human macrophages were setup with identical corresponding treatments but without MTB infection.

### RNA isolation and Quantification

RNA was isolated from macrophages with the mirVana miRNA Isolation Kit (Ambion, Austin, TX, USA) in accordance with the manufacturer’s instructions. The purity and quantity of RNA were measured by NanoDrop (ND-1000 spectrophotometer, Thermo Scientific, Wilmington, DE, USA). The samples were used immediately or stored at -80°C.

### TaqMan microRNA Array Quantitative PCR

The TaqMan MicroRNA Reverse Transcription Kit (Applied Biosystems, Foster City, CA, USA) was used for preparation of cDNA. RT reactions were performed on a GeneAmp PCR System 9600 (Applied Biosystems) with the following conditions: 40 cycles of 16°C, 2 min; 42°C, 1 min; 50°C, 1 sec; and 1 cycle of holding at 85°C, 5 minutes. All samples were analysed with the Human TaqMan low density miRNA array (TLDA, Applied Biosystems) which covered 384 different miRNAs simultaneously and performed using a fast real-time PCR system (ABI Prism 7900HT). The cycle threshold (Ct) raw data was analyzed by two manufacturer’s softwares; SDS 2.4 and RQ Manager 1.2.1. The uninfected control results were set as the baseline against the infected in the analyses.

### Analysis of potential mRNAs targeted by differentially expressed microRNAs

Family names were specified by miRBase release 19, while clustered microRNA described in miRBase release 19 were assumed to be polycistronic pri-miRNAs. Possible mRNA targets of the differentially expressed miRNA were identified by using the miRwalk databases [16], through an integrative evaluation with different algorithms: DIANA-mT (http://diana.cslab.ece.ntua.gr/), miRanda (http://www.microrna.org/microrna/home.do), miRDB (http://mirdb.org/miRDB/), RNA22(http://cbcsrv.watson.ibm.com/rna22.html), and TargetScan v 6.2 (http://www.targetscan.org/). Only mRNA predicted by at least three of these algorithms were considered as potential targets. Cellular pathway analysis of the differentially expressed miRNAs was performedby using the DIANA-miRPath v2.0 [17], based on information from DIANA-microT-CDS (http://diana.cslab.ece.ntua.gr/micro-CDS/?r=search) and the KEGG pathway database (http://www.genome.jp/kegg/pathway.html).

### Statistical analysis

The expression level of each miRNA was calculated by the relative quantity (RQ value) (2^-ΔΔCt^) method. The internal control, Mammal U6, was selected for normalization across all experiments. The Ct raw data were determined by using an automatic baseline and a threshold of 0.2(RQ Manager, ABI, Life Technologies). Significant differences were evaluated in SPSS (v20.0 for Windows). Only miRNAs with a P value of ≤0.05 and with consistent expression in all of the samples were considered as differentially expressed. Unsupervised clustering analysis, using DataAssist v 3.01 of ABI (Life Technologies), was performed to identify the different sub-groups defined by miRNA expression profiles. "The data discussed in this publication have been deposited in NCBI's Gene Expression Omnibus [18] and are accessible through GEO Series accession number GSE65810 for the human miRNA and GSE65811 for THP-1 cells miRNA profiles (http://www.ncbi.nlm.nih.gov/geo/query/acc.cgi?acc=GSE810; http://www.ncbi.nlm.nih.gov/geo/query/acc.cgi?acc=GSE811) respectively.

## Results

### 1. MicroRNA expression profiling of THP-1macrophages infected with MTB strains

**Fig 1:**
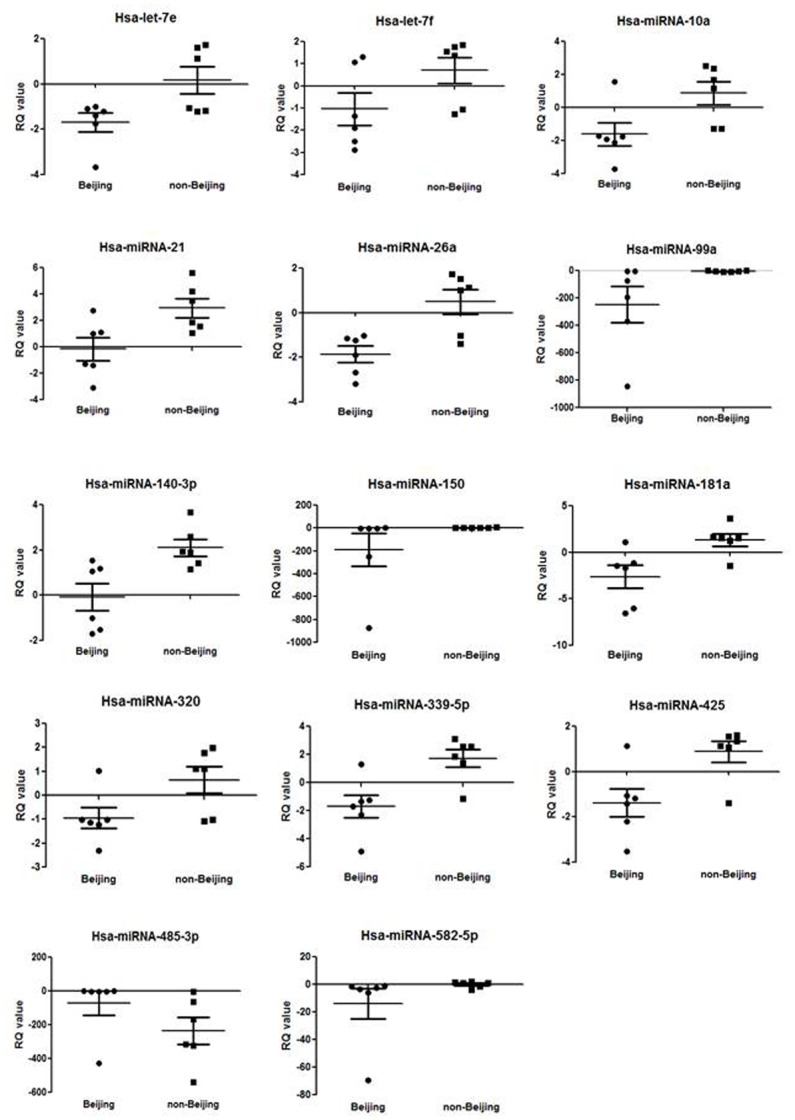
miRNAs expression level in the THP-1 macrophages infected with Beijing/W and non-Beijing/W clinical TB strains. The relative quantity (RQ, 2-ΔΔCt) was used to normalize the relative gene expression data. Statistical analysis between two groups was performed using Mann-Whitney test. Individual values were denoted by black dots/squares from each group of Beijing/W versus non-Beijing strains. The mean RQ and S.D. of each group were represented by the ------- bar and short bars --- in each figure, respectively.

PMA-induced THP-1 macrophages were infected separately with six Beijing/W and six non-Beijing/W strains. MicroRNAs were quantitated by RT-PCR using the Human TaqMan Low Density Array (TLDA). Of the miRNAs that were fully expressed in all samples, statistically significant differential expression (p < 0.05) of 14 miRNAs in macrophages of Beijing/W MTB infection were identified when compared with that of non-Beijing/W strains (Table 1). Of these, 13 miRNAs (hsa-let-7e, hsa-let-7f, hsa-miR-10a, hsa-miR-21, hsa-miR-26a, hsa-miR-99a, hsa-miR-140-3p, hsa-miR-150, hsa-miR-181a, hsa-miR-320, hsa-miR-339-5p, hsa-miR-425, and hsa-miR-582-5p) were repressed in the Beijing/W TB infected group (Fig 1). The cluster analysis is shown in S1 Fig.

**Table 1:** MicroRNAs differentially expressed in THP-1 macrophages infected with Beijing/W and non-Beijing/W clinical TB strains.

	miRNA family	Polycistronic miRNA Precursor	Ratio^a^	P-value^b^
hsa-let-7e	let-7	hsa-mir-99b/hsa-let-7e/hsa-mir-125a	-1.65	0.041
hsa-let-7f	let-7	N/A	-1.87	0.026
hsa-miR-10a	miR-10	N/A	-2.35	0.015
hsa-miR-21	miR-21	N/A	-2.65	0.025
hsa-miR-26a	miR-26	N/A	-1.83	0.015
hsa-miR-99a	miR-99	hsa-let-7c/hsa-miR-99a	-4.34	0.026
hsa-miR-140-3p	miR-140	N/A	-2.10	0.015
hsa-miR-150	N/A	N/A	-8.01	0.002
hsa-miR-181a	miR-181	hsa-miR-181a/hsa-miR-181b	-2.85	0.015
hsa-miR-320	miR-320	N/A	-1.55	0.026
hsa-miR-339-5p	miR-339	N/A	-3.03	0.004
hsa-miR-425	miR-425	hsa-miR-191/hsa-miR-425	-1.70	0.041
hsa-miR-485-3p	miR-485	hsa-miR-381/hsa-miR-487b/hsa-miR-539/hsa-miR-889/hsa-miR-544a/hsa-miR-655/hsa-miR-487a/hsa-miR-382/hsa-miR-134/hsa-miR-668/hsa-miR-485/hsa-miR-323b/hsa-miR-154/hsa-miR-496/hsa-miR-377/hsa-miR-541/hsa-miR-409	14.62	0.041
hsa-miR-582-5p	miR-582	N/A	-2.90	0.041

^a^Fold difference in miRNA expression in THP-1 cells infected with Beijing/W clinical strains vs non-Beijing/W strains.

^b^P-value was calculated by Mann-Whitney test.

Based on these significantly altered miRNA profiles, a number of biological processes were highlighted in infection with different MTB strains. The pathway analyses of miRNA profiles induced by Beijing/W versus non-Beijing/W strains (Table 2) showed that immune-regulatory interactions of the TGF-β signalling pathway were involved. In particular, a change of pathways leading to cell communication (Gap junction, focal adhesion, and adherens junction) and cellular process (endocytosis and apoptosis), as well as signal transduction through MAP kinases, mTOR, ECM receptor, and Wnt were implicated.

**Table 2:** Biological pathways potentially affected by the differentially expressed microRNAs in THP-1 macrophages infected with Beijing/W and non-Beijing/W TB clinical strains.

KEGG pathway	p-value	#genes	Description
TGF-beta signalling pathway	1.60E-05	31	Regulate cell differentiation, proliferation, migration and apoptosis
Wnt signalling pathway	2.07E-05	50	Required for developmental processes: cell-fate specification, cell proliferation and cell division
Lysine degradation	3.25E-05	15	Amino acid metabolism
ECM-receptor interaction	6.05E-05	22	Control of adhesion, migration, differentiation, proliferation, and apoptosis
mTOR signalling pathway	0.000931	22	Signal transduction
T cell receptor signalling pathway	0.002746	37	Activation of T lymphocytes proliferation, cytokine production and differentiation into effector
MAPK signalling pathway	0.003241	74	Involved in various cellular functions: cell proliferation, differentiation and migration
Cytokine-cytokine receptor interaction	0.003241	60	Engaged in host defenses, cell growth, differentiation, cell death, angiogenesis, development and repair processes
Adherens junction	0.004389	25	Important for maintaining tissue architecture and cell polarity and can limit cell movement and proliferation
Protein processing in endoplasmic reticulum	0.004952	46	Newly synthesized peptides glycosylated.
Glycosaminoglycan biosynthesis –heparansulfate	0.005768	11	Cell membrane and extracellular matrix component biosynthesis
Insulin signalling pathway	0.011156	41	Activation of glycogen synthesis and gene transcription
Endocrine and other factor-regulated calcium reabsorption	0.013969	15	Calcium (Ca2+) homeostasis
Apoptosis	0.014	25	Program cell death
Gap junction	0.014	24	Contain intercellular channels that allow communication between the cytosolic compartments of adjacent cells
Adipocytokine signalling pathway	0.017781	22	Positively correlated with leptin production, and negatively correlated with production of adiponectin
Cysteine and methionine metabolism	0.024674	10	Amino acid synthesization
Glycosaminoglycan biosynthesis –keratansulfate	0.036004	5	Glycan biosynthesis and metabolism
Osteoclast differentiation	0.036168	36	Responsible for bone resorption
Fc gamma R-mediated phagocytosis	0.044864	27	An essential role in host-defense mechanisms through the uptake and destruction of infectious pathogens

### 2. MicroRNA Expression in host macrophages of active MTB, latent infection and healthy controls

**Fig 2:**
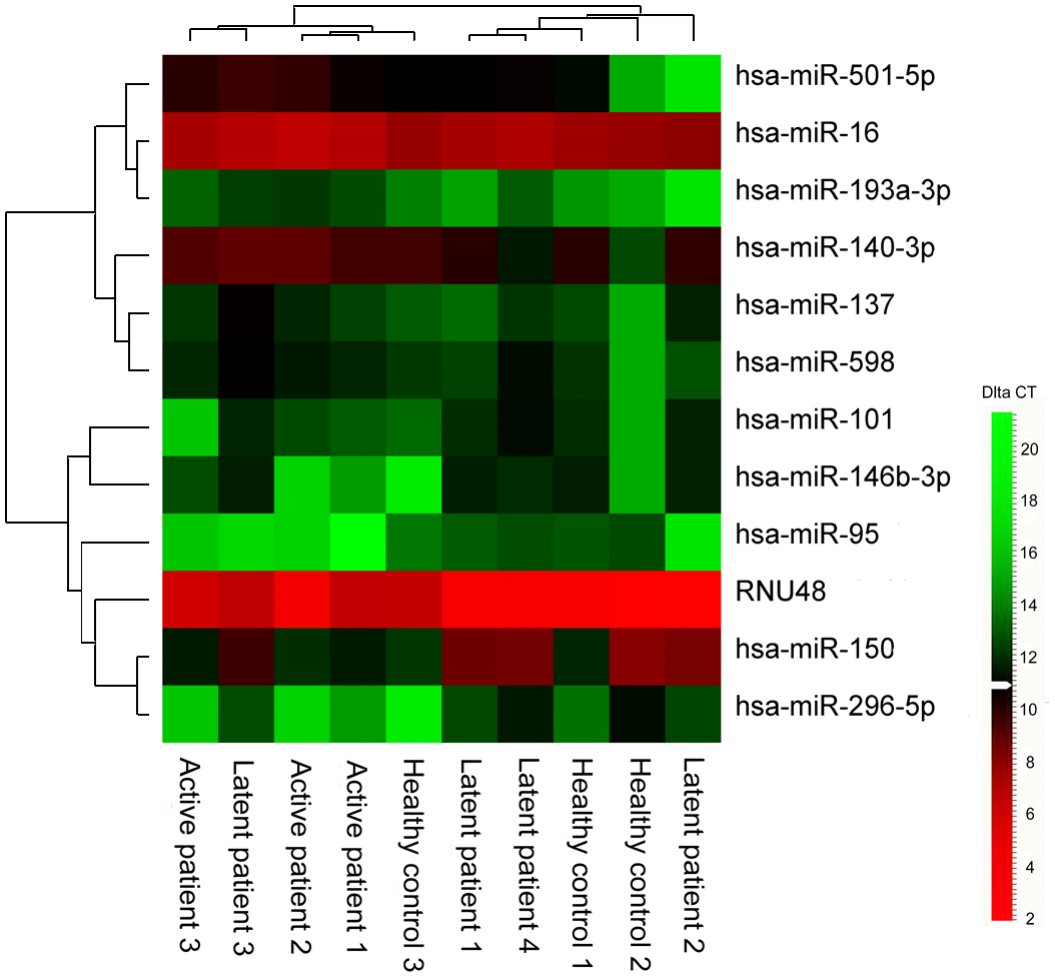
Clustering analysis of the 11 miRNAs was performed using DataAssist 3.0v based on ΔCt-values of the TLDA results. Upregulated miRNAs are designated by various shades of red and down-regulated miRNAs by various shades of green. Clinical phenotypes are labelled in different colours: active MTB infection (red), latent infection (blue), and healthy controls (green).

The miRNA expression in macrophages of active MTB (n = 3), LTBI infection (n = 4), and healthy controls (n = 3) were examined. Details of the subjects are listed (S1 Table). Eleven miRNAs was found to be differentially expressed in the active MTB versus the latent/healthy controls (p < 0.05) (Table 3). Among these 11 miRNAs, no differences were observed between the latent and healthy controls groups. Seven miRNAs had different expression levels between active TB and healthy controls: six miRNAs (hsa-miR-16, hsa-miR-137, hsa-miR-140-3p, hsa-miR-193a-3p, hsa-miR-501-5p, and hsa-miR-598) were upregulated while hsa-miR-95 was down-regulated. Two miRNAs (hsa-miR-101 and hsa-miR-150) were found to differentiate the LTBI group from the MTB active disease group (S2 Fig). Interestingly, hsa-miR-146b-3p and hsa-miR-296-5p were expressed in all of LTBI group but not in the active MTB and healthy controls. Fig 2 shows a tendency for these 11 differentially expressed miRNAs to cluster independently the groups of active MTB disease and the LTBI or healthy controls.

**Table 3:** miRNAs differentially expressed in human macrophages with active MTB and latent infections against healthy controls.

	miRNA family	Polycistronic miRNA Precursor	Latent^a^	Active^b^	P-value^c^
hsa-miR-16	miR-15	miR-15a/miR-16-1	1.38	2.02	0.05
hsa-miR-95	miR-95	N/A	-1.69	-19.01	0.05
hsa-miR-101	miR-101	hsa-miR-3671/hsa-miR-101-1	3.17	-1.34	0.026
hsa-miR-137	miR-137	hsa-miR-2682/hsa-miR-137	5.25	4.66	0.05
hsa-miR-140-3p	miR-140	N/A	1.63	3.79	0.032
hsa-miR-150	N/A	N/A	1.20	-17.79	0.026
hsa-miR-193a-3p	miR-193	N/A	4.14	7.27	0.05
hsa-miR-501-5p	miR-500	hsa-miR-532/hsa-miR-188/hsa-miR-500a/hsa-miR-362/hsa-miR-501/hsa-miR-500b/hsa-miR-660/hsa-miR-502	2.40	5.12	0.05
hsa-miR-598	miR-598	N/A	2.75	3.58	0.05
hsa-miR-146b-3p	miR-146	N/A			N/A
hsa-miR-296-5p	miR-296	hsa-miR-296/hsa-miR-298			N/A

^a^Indicates miRNA expression in macrophages of latent group vs healthy controls.

^b^Indicates miRNA expression in macrophages of active group vs healthy controls.

^c^P-value was obtainedby an independent median test.

The biological pathways potentially implicated by these differentially expressed miRNAs are listed in Table 4. Pathway analyses identified that the change of cell membrane and extracellular matrix metabolite involving glycosaminoglycan biosynthesis-HS and fatty acid biosynthesis might play a role in MTB infection. This might result in signal transduction through MAP kinases, mTOR, ECM receptor, and Wnt, and finally activate the immune-regulatory interactions involving the TGF-β signalling pathway and the T cell receptor signalling pathway.

**Table 4:** Biological pathways potentially affected by the differentially expressed microRNAs of significance from macrophages of active MTB disease, LTBI and healthy controls.

KEGG pathway	p-value	# of genes	Description
Glycosaminoglycan biosynthesis –heparansulfate	6.02E-28	4	Cell membrane and extracellular matrix component biosynthesis
Fatty acid biosynthesis	8.58E-15	1	Lipid Metabolism
MAPK signalling pathway	0.000271	59	Involved in various cellular functions: cell proliferation, differentiation and migration
Wnt signalling pathway	0.004258	38	Required for developmental processes: cell-fate specification, cell proliferation and cell division
Ubiquitin mediated proteolysis	0.005826	33	Functions as a signal for 26S proteasome dependent protein degradation
Insulin signalling pathway	0.006315	33	Activation of glycogen synthesis and gene transcription
VEGF signalling pathway	0.008692	21	A crucial signal transducer in both physiologic and pathologic angiogenesis
Circadian rhythm—mammal	0.011601	8	An internal biological clock to sustain the absence of environmental cues
Oocyte meiosis	0.011601	24	Involved in cell growth and death
TGF-beta signalling pathway	0.011601	18	Regulate cell differentiation, proliferation, migration and apoptosis
ErbB signalling pathway	0.012265	23	Regulate proliferation, differentiation, cell motility and survival
Focal adhesion	0.013426	42	Cell-matrix adhesions
Phosphatidylinositol signalling system	0.015405	16	An important intracellular second-messenger signaling system
Notch signalling pathway	0.016696	14	Essential for proper embryonic development in all metazoan organisms
T cell receptor signalling pathway	0.016696	26	Activation of T lymphocytes proliferation, cytokine production and differentiation into effector cells
Endocytosis	0.016696	42	Bring ligands, nutrients, plasma Membrane proteins and lipids from the cell surface into the cell interior

## Discussion

MiRNAs can modulate the innate and adaptive immune responses to pathogens by affecting mammalian immune cell differentiation and the development of diseases of immunological origin [19], because various bacterial cell wall components, such as peptidoglycan (PG), lipoproteins and lipopolysaccharide (LPS) could upregulate the miRNAs levels [20].

In our studies, miRNA profiles in the macrophages were found to be altered in MTB infection in a strain- and host-dependent way. The Beijing genotype strain is the most predominant *M*. *tuberculosis* strain in south China, and it has caused large outbreaks of MDR-TB. The Beijing strains showed increased transmission fitness when they acquired streptomycin resistance [21]. Beijing genotype strains were also found to induce the STAT1 activation and interferon-related immune response [22–23]. We showed for the first time that the Beijing/W strains repressed a number of miRNAs as compared to the non-Beijing/W TB strains, which might reflect their virulence characteristics in altering the host response. Hsa-miR485-3p was found to be upregulated in Beijing/W infected macrophages. Hsa-miR-485-3p has been shown to be involved in cell survival [24] and knockdown of this miRNA in hepatic cells increased apoptosis [25]. Previous report indicated that miR-485-3p post-transcriptionally targeted NF-YB [24], a direct transcriptional repressor of Top2α gene and of MDR1 and CCNB2 genes [26] in regulation of the cell cycle, Our results suggest that high miR-485-3p possibly facilitates survival of the Beijing/W strains in macrophages and evades apoptosis or alters macrophage lysis and subsequent downstream immune response toward clearance of MTB.

The difficulty in discriminating the spectrum of MTB infections and of latency is prompting the need to search for new biomarkers for MTB infection. Previous studies that have utilized such microarrays as diagnostic markers are listed in Table 5. Studies used whole-genome transcriptional profiling of peripheral blood mononuclear cells (PBMCs) [27] or whole blood cells [28] found that FcGR1B (CD64) and Fc gamma receptor 1B (FCGRIB) were the most differentially expressed genes in the individuals with active TB. A recent report found a dominant TNF-a+ MTB–specific CD4+ T cell response that discriminated between LTBI and active disease [29]. The miRNA expression profile of PBMCs [30] and sputum supernatant [31] exhibited a characteristic expression in MTB infection, while the miRNA signatures from serum also associated to different phases of TB infections [32–35]. These data may shed some light to the roles of miRNAs in MTB infections, but do not yet explain the transition of latency to active TB disease. We were able to distinguish with the expression of 11-miRNA signature profiles of the active TB group from that of the LTBI group but not that of latent and healthy groups. When we carried out the analysis using group-wise comparisons, the variations between individual group members showed that 10–25% of the latent patients remained clustered with the active TB patients, and this corroborated with a previous study which concluded that the whole-blood transcript dominated by neutrophil-driven interferon (IFN)-inducible genes correlated with the radiological extent of active MTB [36].

**Table 5:** Potential biomarkers for latent and active TB infections based on miRNA or whole genome microarray studies.

Test groups	Sample	Array type	Finding	Reference
TB active, latent and normal	Whole blood	Whole-genome oligonucleotide microarray (Agilent Technologies)	Fc gamma receptor 1B (FCGRIB)	[28]
H37Rv orΔ-mce1 H37Rv bacteria	Murine macrophages	Oligo whole-mouse Genome microarrays (Agilent Technologies)	Mce1 protein complex	[37]
Active TB, LTBI, and Healthy Control	PBMCs	The Agilent Human miRNA microarray platform	Different pathways	[30]
Active TB, LTBI, and Healthy Control	PBMCs	Agilent custom designed oligonucleotide microarrays	CD64	[27]
Active TB and Healthy Control	PBMCs	Agilent’s human miRNA microarray	miR-155	[12]
Active TB and Healthy Control	serum	miRCURY LNA array (Exiqon)	miR-29a	[11]
Active TB and Healthy Control	PBMCs	miRCURY LNA microRNA array (Exiqon)	miR-144*	[6]
Healthy donor infected with M. avium subsp. hominissuis	PBMC derived macrophages	miRCURY LNA microRNA array (Exiqon)	Let-7e, miR-29a, miR-886-5p	[38]
Active TB and Healthy Control	Sputum	miRCURY LNA microRNA array (Exiqon)	miR-19b-2*, miR-3179, miR-147	[31]
Beijing strain & latent MTB strain	Rabbit lung	Whole genome rabbit microarray (Agilent)	Inflammatory response and STAT1 activation	[22]
Beijing MTB strain	THP-1 cell	HG-U133 Plus 2.0 array (Affymetrix)	Interferon-related immune response	[23]
PTB, EPTB, LTBI	serum	Taqman low density array (TLDA, Life Technologies)	10 miRNA profile for European group; 12 miRNA profile for African group	[33]
PTB	serum	Taqman low density array (TLDA, Life Technologies)	miR-361-5p, miR-889, miR-576-3p	[32]
Active TB, LTBI, and Healthy Control	Whole blood	Illumina human HT-12 beadchip array	Neutrophil-driven IFN-inducible gene profile	[36]
H37Rv	Murine dendritic cell	miRCURY LNA microRNA array (Exiqon)	miR-99b, miR-146a, miR-125a-5p	[8]
H37Rv	RAW264.7	SYBR Green-based miRNA profiling array (SA Biosciences)	Let-7f	[39]
H37Rv	PBMC-derived macrophage from healthy donor	TaqMan Low-Density Array v2.0 (Applied Biosystem, CA, USA)	miR-155,miR-146a, miR-145,miR-222*, miR-27a, miR-27b	[40]
H37Rv and H37Ra	THP-1 macrophages	Microarray from commercial provider ‘LC Sciences’, USA	miR-30a, miR-30e, miR-155, miR-1275, miR-3665, miR-3178, miR-4484, miR-4668-5p and miR-4497	[41]

The microRNA profile in the human macrophage was quite different from that of whole blood, sputum and PBMCs from the literature. In our study, some of the miRNAs were proven to play key roles in the immune and inflammatory pathways, and their biological targets in MTB infection have been previously described (Table 6). The miRNA-146 family was found to play key roles in the anti-inflammatory reaction. miR-146b could be induced by LPS or PG from bacteria [42]. miR-146a/b was a negative regulator of constitutive NF-kB activity, which results in the suppression of IL-1 receptor-associated kinase 1 and TNF receptor-associated factor 6 protein levels [42,43]. In our study, the expression of miR-146b in the LTBI group was significantly higher than that in active TB infections. We propose that hsa-146b-3p may be highly related to the LTBI.

**Table 6:** Previously reported microRNAs with differential expression related to current study of MTB infections and their validated transcript targets.

microRNA name	Validated target	Reference
hsa-miR-146b-3p	IL1B, IL6, IRAK1, TRAF6, TNF, TLR4, NFKB1 and MMP16	[42–43]
hsa-miR-21	PTEN, hSulf-1, PDCD4, IL-12p35 and Bcl-2	[44–47]
hsa-miR-150	NOTCH3, c-myb	[48–51]
hsa-miR-101	RC3H1, IL10, IL17A, IL17D	[52]
hsa-miR-140-3p	CD38	[53]
hsa-miR-181a	K-ras, MAP2K1, MAPK1 and Snai2	[54, 55]
hsa-miR-26a	IL6, CDC6, PRL-1	[56–58]
hsa-miR-193a-3p	Mcl-1, ERBB4, S6K2	[59,60]
hsa-miR-296-5p	BAX, Bcl2, MDR1, CyclinD1, P27	[61]

miR-21 can be induced after Bacillus Calmette-Guerin (BCG) vaccination by NF-kB activation. miR-21 suppressed the IL-12 production by targeting IL-12p35, which impaired anti-mycobacterial T cell responses both *in vitro* and *in vivo*. Additionally, miR-21 also promoted dendritic cell (DC) apoptosis by targeting Bcl-2. Therefore, miR-21 may potentially be involved in the fine-tuning of the anti-mycobacterial Th1 response and in regulating the efficacy of BCG vaccination [44–47].

miR-150 has been one of the extensively studied miRNAs, and it has been demonstrated to be selectively expressed in mature naive B and T cells, being down-regulated in their progenitors or in lymphocyte activation and strongly upregulated as maturation progresses [48–51]. The well-known targets for miR-150 are NOTCH3 (a member of the Notch receptor family) and c-Myb (a transcription factor that plays an essential role in the hematopoietic process that plays important roles both in T-cell differentiation and leukemogenesis). In our study, the Beijing/W clinical strains suppressed the miR-150 and miR-21expression and they may play a role in virulence. Lower expression of miR-150 in the active TB patients compared with the latent and healthy controls may be due to the reduced mature T cells and B cells in patients with active TB, as previous studies have shown [36].

Both miR-150 and miR-140-3p were differentially expressed in macrophages infected in vitro and those from active TB patients. These two miRNAs are related with the secondary signal transduction pathway, which and likely involved in MTB infection. Four predicted pathways, including Wnt signalling pathway, insulin signaling pathway, TGF-β signalling pathway and glycosaminoglycan biosynthsis, are involved in Beijing/W & non-Beijing/W (Table 2) and active MTB& LTBI (Table 4) studies. This reaffirms the involvement of the inflammatory defence and signal transduction and cell communication in the macrophages in in MTB infection in vitro and in the host.

Four pathways of cell membrane and communication (adherens junction, gap junction, glycosaminoglycan biosynthsis-heparan sulfate/keratin sulfate metabolite), suggesting that Beijing/W TB strain may affect macrophage survival by altering their cell membrane structure and limit the downstream host immunological defence reaction.

The inflammatory miRNA miR-146b-3p, miR-101 and the cell survival miRNA miR-193a-3p and miR-296-5p were only found differentially expressed in macrophages of active TB group, suggesting response that alters macrophage survival in the infected host.

In addition, compared with whole blood, the microRNA profile revealed from the adherent human macrophages reflect the molecular changes in the TB-engulfed macrophages, bringing insights into the immunological defence mechanisms of these macrophages, where the initial clearance of MTB takes place during infection. On the contrary, the microRNA profiles of blood are the orchestrated outcome of all inflammatory cells and their immune mediators in the host-bacterial interaction, not simply MTB infection of a single immune cell type [11, 28, 32, 33]. Differentially expressed miRNAs and their transcriptional targets might potentially affect the regulation of multiple biological networks. Pathway analysis of our expression profile determined different transcripts that were modified by these miRNAs. These results provide clues for the identification of transcriptionally regulated mechanisms of key biological processes in TB, enhance our understanding of the fundamental biology of MTB, and offer leads for new diagnostics in the future.

## Supporting Information

S1 Fig Clustering analysis of the 16 miRNAs was performed using DataAssist 3.0v based on ΔCt-values of the TLDA results. Upregulated miRNAs are designated by various shades of red and down-regulated miRNAs by various shades of green.
(TIF)

S2 Fig miRNA expression levels in human macrophages with LTBI, active MTB disease and in healthy controls. Statistical analysis between two groups was performed using the unpaired t-test. Individual values were denoted by black dots/squares/triangles from each group. The mean RQ and S.D. of each group were represented by the ------- bar and short bars --- in each figure, respectively.
(TIF)

S1 Table Characteristics of active TB, latent and healthy controls in this study. (DOCX)
