# Supplementary figures and images for: Differential MicroRNA Expression in Human Macrophages with Mycobacterium tuberculosis Infection of Beijing/W and Non-Beijing/W Strain Types
Source: PLoS One. 2015 Jun 8;10(6):e0126018. doi: 10.1371/journal.pone.0126018 (PMC4460131; doi:10.1371/journal.pone.0126018)

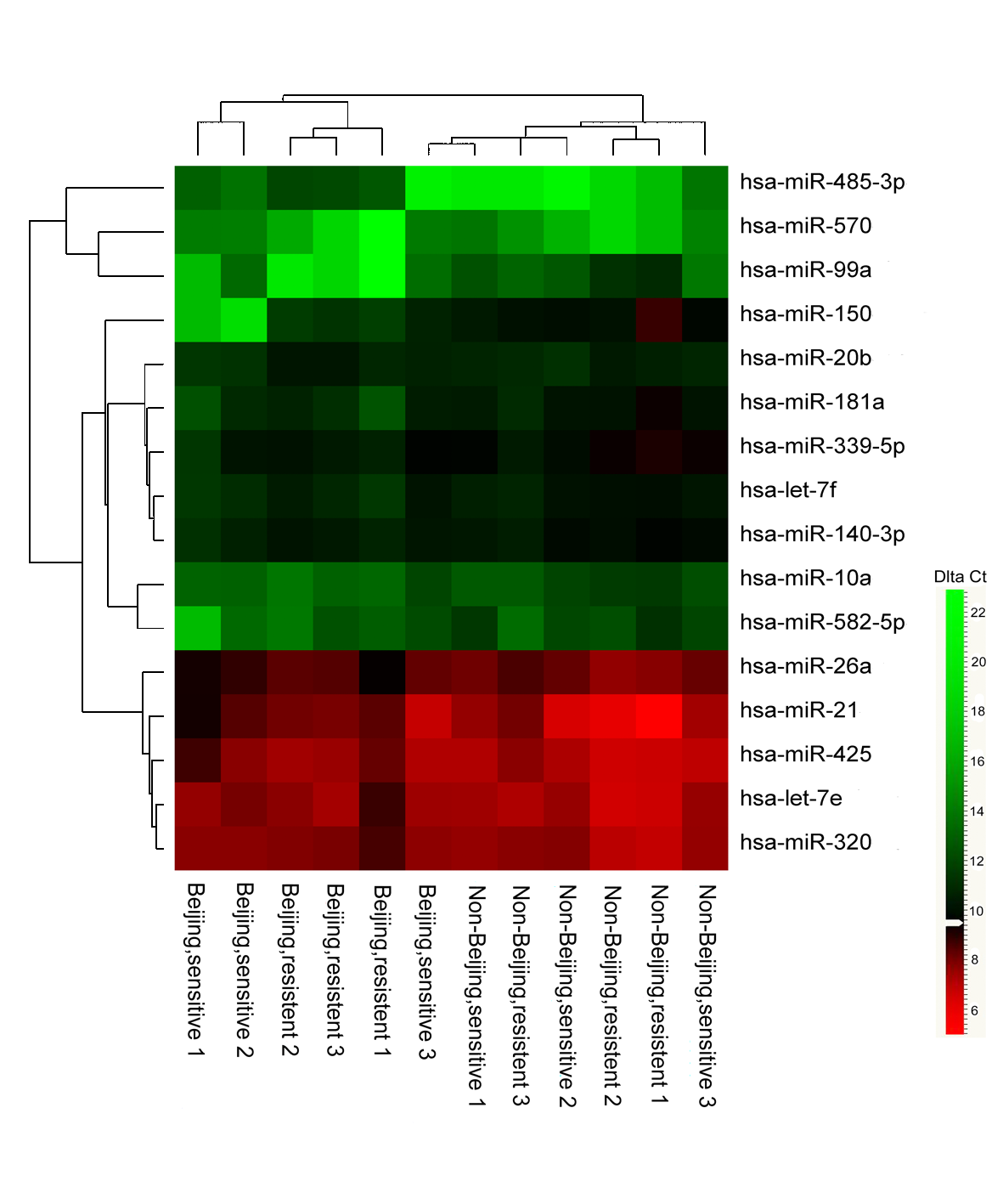

Supplement: S1 Fig — Upregulated miRNAs are designated by various shades of red and down-regulated miRNAs by various shades of green. (TIF) [file pone.0126018.s001.tif]

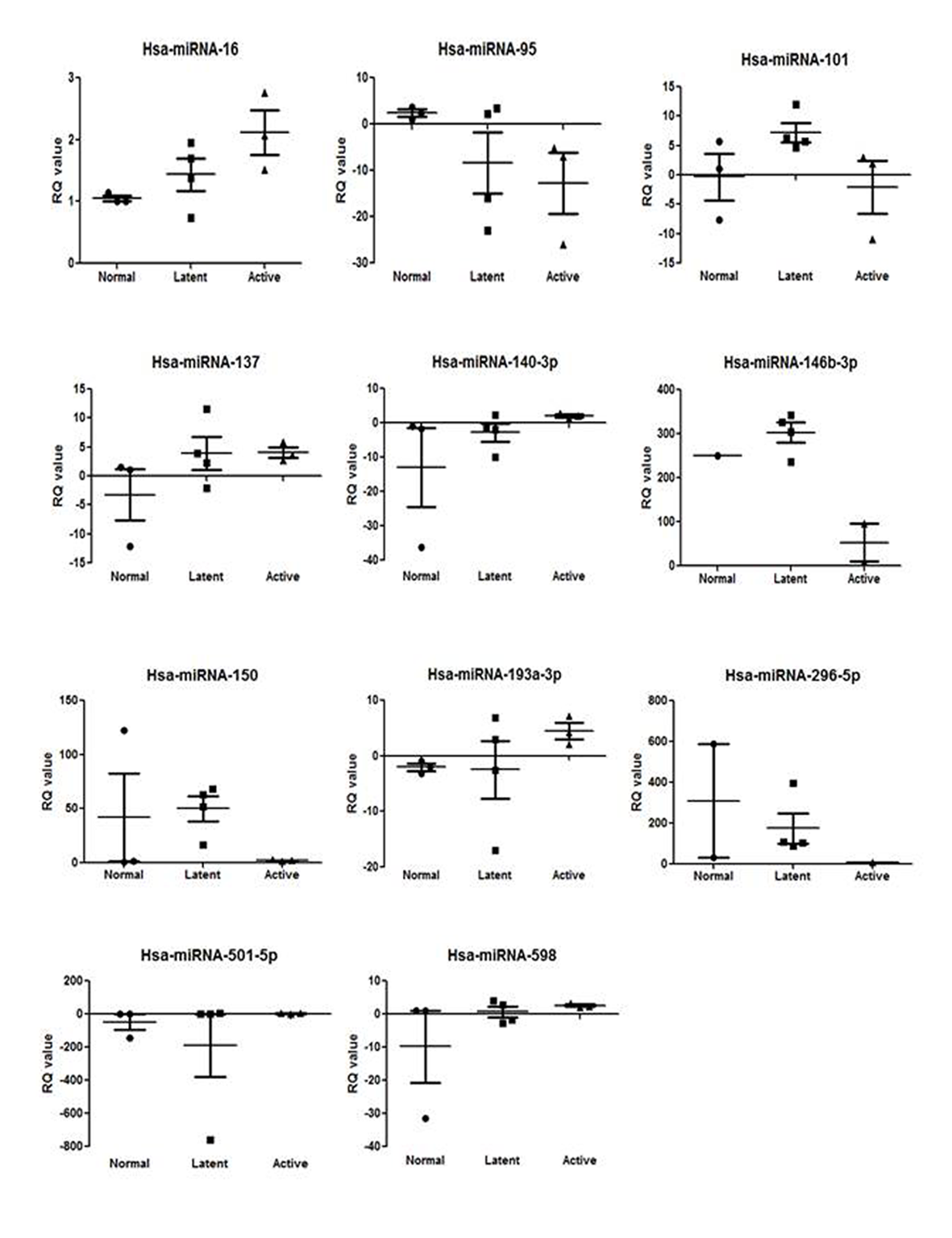

Supplement: S2 Fig — Statistical analysis between two groups was performed using the unpaired t-test. Individual values were denoted by black dots/squares/triangles from each group. The mean RQ and S.D. of each group were represented by the ------- bar and short bars --- in each figure, respectively. (TIF) [file pone.0126018.s002.tif]
