# Supplementary material for: Differential MicroRNA Expression in Human Macrophages with Mycobacterium tuberculosis Infection of Beijing/W and Non-Beijing/W Strain Types
Source: PLoS One. 2015 Jun 8;10(6):e0126018. doi: 10.1371/journal.pone.0126018 (PMC4460131; doi:10.1371/journal.pone.0126018)
Supplement: S1 Table — (DOCX) [file pone.0126018.s003.docx]

**Supporting information S1 Table:**

**S1 Table. Characteristics of active TB, latent and healthy controls in this** **study**

| **Group** | **Age** | **Sex** | **IGRA^a^** |
| --- | --- | --- | --- |
| Acute 1 | 57 | M | Positive |
| Acute 2 | 27 | F | Positive |
| Acute 3 | 90 | M | Positive |
| Latent 1 | 42 | M | Positive |
| Latent 2 | 58 | M | Positive |
| Latent 3 | 57 | F | Positive |
| Latent 4 | 42 | M | Positive |
| Normal 1 | 52 | M | Negative |
| Normal 2 | 23 | M | Negative |
| Normal 3 | 34 | F | Negative |

^a^: IGRA test was performed by the QuantiFERON TB-Gold Test
